# Dithieno[3,2-*b*:2′,3′-*d*]silole-based conjugated polymers for bioimaging in the short-wave infrared region†

**DOI:** 10.1039/d1ra05097d

**Published:** 2021-09-16

**Authors:** Chuantao Gu, Haicheng Wang, Xiaoxia Wang, Shuguang Wen, Xiaoguang Liu, Weiqiang Tan, Meng Qiu, Jiping Ma

**Affiliations:** School of Environmental and Municipal Engineering, Qingdao University of Technology Qingdao 266525 P. R. China guchuantao@qut.edu.cn +86-532-85071673; CAS Key Laboratory of Bio-based Materials, Qingdao Institute of Bioenergy and Bioprocess Technology, Chinese Academy of Sciences Qingdao 266101 P. R. China; Key Laboratory of Marine Chemistry Theory and Technology (Ocean University of China), Ministry of Education Qingdao 266011 P. R. China mengqiu@ouc.edu.cn; Qing Dao Municipal Hospital Qingdao 266011 P. R. China

## Abstract

The short-wave infrared window (SWIR, 900–1700 nm) fluorescence imaging has been demonstrated to have excellent imaging performance in signal/noise ratio and tissue penetration compared to the conventional NIR biological window (NIR-I, 700–900 nm). Conventional organic SWIR fluorescent materials still suffer from low fluorescence quantum efficiency. In this work, a donor unit with sp^3^ hybrid configuration and an acceptor unit with small hindered alkyl side chains are employed to construct donor–acceptor (D–A) type conjugated polymers P1 and P2, which were substituted with one or two fluorine atoms. These structural features can alleviate the aggregation-caused quenching (ACQ) and contribute to charge transfer, resulting in a significantly improved fluorescence quantum efficiency. The SWIR fluorescent quantum efficiencies of P1 and P2 nanoparticles are 3.4% and 4.4%, respectively, which are some of the highest for organic SWIR fluorophores reported so far. Excellent imaging quality has been demonstrated with P2 nanoparticles for SWIR imaging of the vascular system of nude mice. The results indicate that our design strategy of introducing sp^3^ hybrid configuration and small hindered alkyl side chains to fabricate conjugated polymers is efficient in improving the fluorescent quantum efficiency as SWIR fluorescent imaging agents for potential clinical practice.

## Abstract 2

A D–A type polymer with a SWIR fluorescence quantum efficiency of 4.4% was obtained after structural optimization.

## Graphical abstract

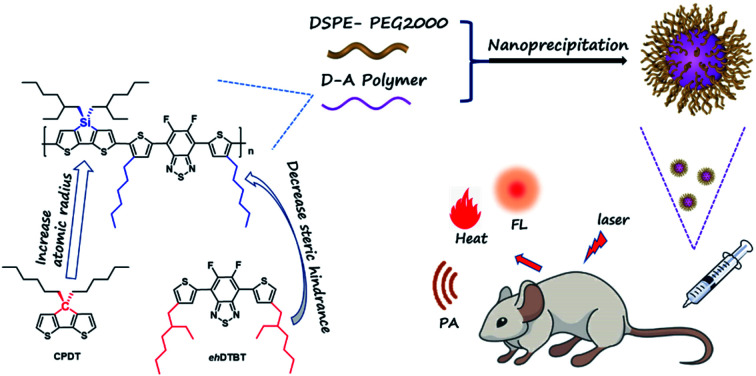

## Introduction

Fluorescence imaging technology with high resolution and high sensitivity is an emerging visualization tool for *in vivo* detection.^1–4^ Compared with the conventional NIR biological window (NIR-I, 700–900 nm), the short-wave infrared window (SWIR, 900–1700 nm) imaging has lower autofluorescence, deeper tissue penetration depths and higher signal-noise ratio.^5–7^ High-performance fluorescent probes are the basis for obtaining high-quality fluorescence imaging. At present, a series of SWIR fluorescent probes based on inorganic and organic materials have been developed, including organic nanoparticles based on conjugated polymers,^8–10^ small molecule dyes,^2,11–14^ as well as inorganic Ag_2_S quantum dots,^15–17^ rare earth doped nanoparticles^18–20^ and single-walled carbon nanotubes,^21–23^*etc.* The lack of good water solubility, stability, biocompatibility and low fluorescence efficiency is the bottleneck for the development of SWIR fluorescent materials.^8,24,25^ The ideal SWIR fluorescent materials should have high absorption coefficient, large Stokes shift, adjustable emission wavelength, high quantum efficiency and good biological safety.^8,26^ Considering these aspects, donor–acceptor (D–A) type conjugated polymers are promising SWIR fluorescent materials. Prof. Hongjie Dai first reported the application of a D–A type conjugated polymer named as pDA as a SWIR fluorescent material in the field of SWIR fluorescence imaging in 2014.^8^ Since then, a large number of D–A type conjugated polymers have been used in biomedical fields such as SWIR fluorescence imaging,^10,27,28^ photothermal therapy,^29–31^ photoacoustic imaging^32–34^ and photodynamic therapy.^35,36^

However, the fluorescence quantum efficiency of D–A type conjugated polymer fluorescent materials still needs to be improved. The polymers have long-chain conjugated structures and are prone to π–π stacking, leading to aggregation-caused quenching (ACQ).^37,38^ The development of aggregation-induced emission (AIE) materials by Prof. Benzhong Tang^3,38,39^ can solve this problem, which is a representative solution. The highest SWIR fluorescence quantum efficiency of AIE material has been higher than 10%.^40^ In recent years, we have tried to solve this problem through non-AIE methods,^27,28,37,41^ rational molecular structure design has been used to alleviate the aggregation of polymers, thereby reducing the ACQ, and then the fluorescence quantum efficiency can be improved. Two strategies had been used to weaken polymer accumulation in our previous work. One is to introduce large sterically hindered side chains to the polymer backbone,^41^ in order to twist the backbone to a certain extent within the range of not affecting the charge transfer and weaken the ACQ, but this range is difficult to control. A conjugated polymer with large steric hindrance 2-ethylhexyl as side chain had been synthesized in our previous work, and its fluorescence quantum efficiency is 0.5%.^41^ The effect of this strategy on the improvement of fluorescence quantum efficiency is not obvious. The second strategy is introducing donor units with sp^3^ hybrid configuration^27,37^ such as 4*H*-cyclopenta[2,1-*b*:3,4-*b*′]dithiophene (CPDT).^37^ The sp^3^ hybrid has a tetrahedral configuration. The plane of the alkyl chain is perpendicular to the plane of the polymer backbone, which can effectively reduce the aggregation of the polymer. The synthesized polymer with CPDT as donor unit has a fluorescence quantum efficiency of 1.7%,^37^ which is a relatively successful strategy.

**Fig. 1:**
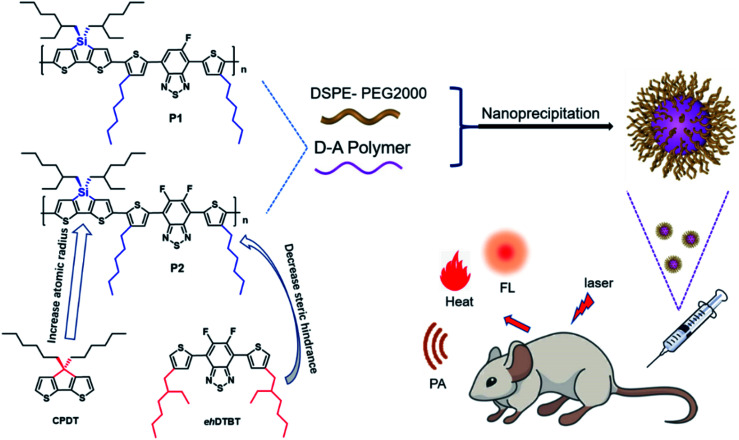
Structure of P1, P2 and schematic illustration of nanoprecipitation.

In this work, two improvements have been made based on the foundation of our previous works. Firstly, the sp^3^ carbon atom in the CPDT unit is replaced by larger silicon atom, which will further amplify the advantages of the sp^3^ hybrid configuration and help to alleviate ACQ. Secondly, the large steric hindrance side chain 2-ethylhexyl is replaced by a small sterically hindered *n*-hexyl as polymer side chain, which is benefit for the planarity of polymer and facilitate charge transport. The hexyl DTBT substituted with one or two fluorine substitute was used as the acceptor unit to synthesize polymer P1 and P2 as shown in the Fig. 1. Nanoparticles of P1 and P2 were prepared through nanoprecipitation. P2 nanoparticles showed an absorption peak at 645 nm and an emission peak at 945 nm, while P1 nanoparticles showed absorption peak at 663 nm and an emission peak at 944 nm. The fluorescence quantum efficiency of P1 and P2 nanoparticles was 3.4% and 4.4%, respectively. The P2 nanoparticles were successfully applied in *in vivo* imaging of vascular system of nude mice. Its photoacoustic and photothermal properties also have been investigated.

## Results and discussion

### Synthesis and characterization

**Fig. 2:**
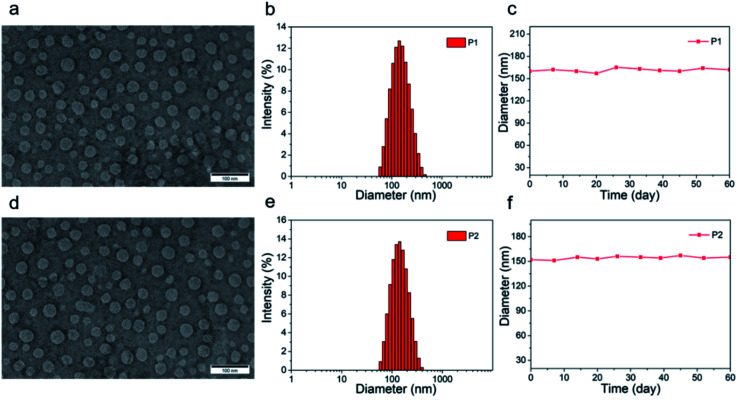
(a and d) TEM picture of nanoparticles of P1 and P2, the scale bar of TEM images is 100 nm; (b and e) size distribution of nanoparticles of P1 and P2, characterized by DLS; (c and f) nanoparticle size of P1 and P2 change within 60 days.

The tetrahedral C (sp^3^) of donor unit was replaced by a tetrahedral Si (sp^3^) atom and the dithieno[3,2-*b*:2′,3′-*d*]silole (DTS) was obtained. The atomic radius of Si is larger than atomic radius of carbon, which could amplify the advantages of sp^3^ configuration. One or two fluorine atoms substituted 4,7-bis(4-hexylthienyl)-2,1,3-benzothiadiazole (DTBT) served as acceptor units, Stille coupling polymerization using a Pd_2_(dba)_3_/P(*o*-tol)_3_ catalytic system between donor unit and acceptor unit gave conjugated copolymers, P1 and P2 (Fig. 1). The yields of both polymers were above 75% and the structure of both polymers was characterized by ^1^H NMR spectroscopy. Detailed synthetic information was described in the ESI.† The amphiphilic phosphoethanolamine-*N*-[methoxy(polyethylene glycol)-2000] (DSPE-PEG2000) was used as the matrix to encapsulate the P1 or P2 to endow the nanoparticles with good biocompatibility and good water solubility (Fig. 1). Transmission electron microscopy (TEM) was employed to characterize the morphology of nanoparticles (Fig. 2a and d). The nanoparticles of P1 and P2 were 20–35 nm in diameter when dehydrated. The hydrated size of nanoparticles was measured by dynamic light scattering (DLS). The results showed the average hydrated diameter of P1 nanoparticles was 160 nm (Fig. 2b) and P2 nanoparticles was 152 nm (Fig. 2e). The zeta potential of P1 and P2 nanoparticles was −2.75 and −3.50 mV, respectively. The nanoparticles were storage at 4 °C and their size was continuously monitored for two months (Fig. 2c and f). Their sizes remained nearly the same, no precipitation was observed for two months, suggesting that their size was relatively stable in water.

### Computational study

**Fig. 3:**
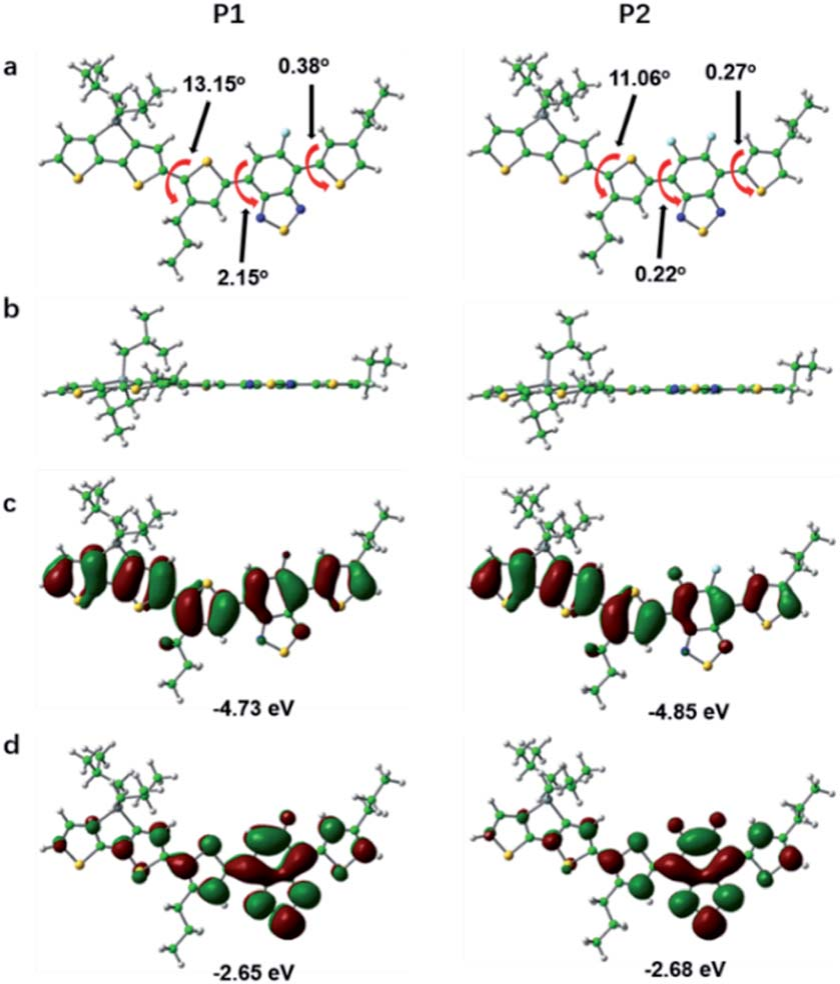
The front view (a), side view (b) of energy minimum conformations and the calculated HOMO (c), LUMO (d) energy levels of P1 and P2. Color code: green (C), white (H), blue (N), yellow (S), grey (Si), light blue (F).

In order to further study the relationship between the fluorescence properties and structures, density functional theory (DFT) at the B3LYP/6-31G(d,p) level^42^ was employed to perform energies and distributions of the frontier molecular orbitals and conformational analysis of P1 and P2. The alkyl chains were simplified to propyl or isobutyl and one repeat unit was chosen in this work in order to reduce calculation time. The energy minimized conformational structures and energies and distributions of the corresponding highest occupied molecular orbitals (HOMOs), lowest unoccupied molecular orbitals (LUMOs) of P1 and P2 are plotted in Fig. 3. The LUMOs are mainly distributed on DTBT units which served as electron acceptor units, while the HOMOs are almost delocalized along the whole backbone, indicating effective intramolecular charge transfer within both polymer backbone to result in long wavelength absorption.^4,29,43^ The fluorine substituent on acceptor unit has big influence on intramolecular or intermolecular interactions.^44^ The noncovalent attractive interaction introduced by fluorine substituent had been demonstrated that it has the function of minimizing torsional angles of polymer backbones.^44,45^ As can be seen from Fig. 3, when compared with P1, the introduction of the second fluorine on acceptor unit of P2 decreases the dihedral angles of the polymer backbone, which can be attributed to the stronger π–π stacking and enhanced F–H, F–F interactions. To be specific, the coplanarity of P2 is better than P1. The better coplanarity could weaken the nonradiative transition and enhance the radiative transition.^43^ Therefore, a higher fluorescence quantum efficiency of P2 can be expected.

### Optical properties

**Fig. 4:**
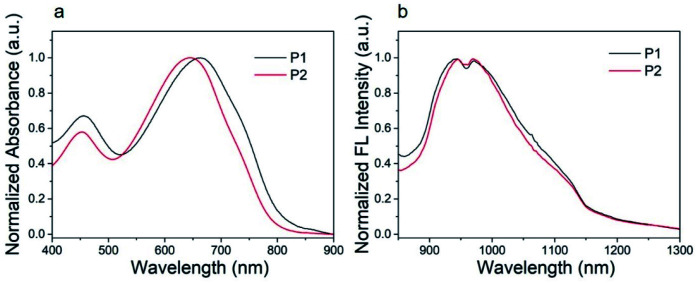
(a) Absorption spectra and (b) fluorescence spectra of P1, P2 nanoparticles in water (100 mg mL^−1^).

The ultraviolet-visible (UV-vis) absorption spectra of P1 nanoparticles and P2 nanoparticles in aqueous solution are shown in Fig. 4. These nanoparticles show similar absorption bands in aqueous solution with major absorption peaks at 663 and 645 nm for P1 and P2, respectively. This characteristic absorption peak is caused by the intramolecular charge transfer (ICT) between the donor units and the acceptor units.^46^ The other absorption peak at about 455 nm can be attributed to the π–π* transition.^46^ As can be seen from Fig. 4, the major absorption peak of P2 was blue shifted when compared with P1. This was caused by the second fluorine atom in P2. The influence of fluorine atom on HOMO is greater than that on LUMO, as a result, the bandgap of P2 was increased when the second fluorine atom was introduced onto the polymer.^47^ The result was consistent with the calculations. The calculated maximum molar extinction coefficient (*ε*) of the diluted P1 and P2 solution (10^−6^ mol L^−1^ in chloroform, Fig. S2†) is 4.22 × 10^5^ L M^−1^ cm^−1^ at 598 nm and 4.95 × 10^5^ L M^−1^ cm^−1^ at 582 nm respectively. Both polymers have strong light absorption, which is beneficial for obtaining high fluorescence quantum efficiency.

The P1 and P2 nanoparticles solution (100 mg mL^−1^) was excited by 808 nm laser to study its fluorescence properties. The normalized fluorescence spectra of P1 and P2 nanoparticles showed an excitation peak at about 945 nm with a tail extended to 1200 nm, as shown in Fig. 4b. Both P1 and P2 nanoparticles shows large Stokes shift more than 280 nm, which is conducive to obtaining high-quality fluorescence imaging. The quantum yield of P1 and P2 was determined to be 3.4% and 4.4%, using IR-26 as a reference with a nominal quantum yield of 0.5%.^8^ Detailed test methods were described in the ESI.† To our best knowledge, the fluorescence quantum efficiency of P1 and P2 is higher than majority of organic SWIR fluorescent materials.^48^ Thus, P1 and P2 are very suitable for *in vivo* imaging. The high quantum efficiency of P1 and P2 should be attributed to their structural characteristics. Firstly, the monomer of DTS with tetrahedral Si (sp^3^) can increase the distance between polymer backbones and reduce the aggregation quenching effect. Secondly, compared with our previous work^41^ the branched alkyl on acceptor unit (2-ethylhexyl) was replaced by a linear hexyl, the dihedral angles of the polymer backbone are reduced and the coplanarity is better, which is conducive to intramolecular charge transfer and helpful to improve the fluorescence quantum efficiency. The detailed absorption and fluorescence data are summarized in Table 1.

**Table 1:** Optical properties of P1 and P2

Polymer	*ε* a (L mol^−1^ cm^−1^)	Absorption peak (nm)	Emission peak (nm)	Stokes shift (nm)	QY (%)
P1	4.22 × 10^5^	663	944	281	3.4
P2	4.95 × 10^5^	645	945	300	4.4

a Molar extinction coefficient data was collected in CHCl_3_ solution.

### Cytotoxicity test

**Fig. 5:**
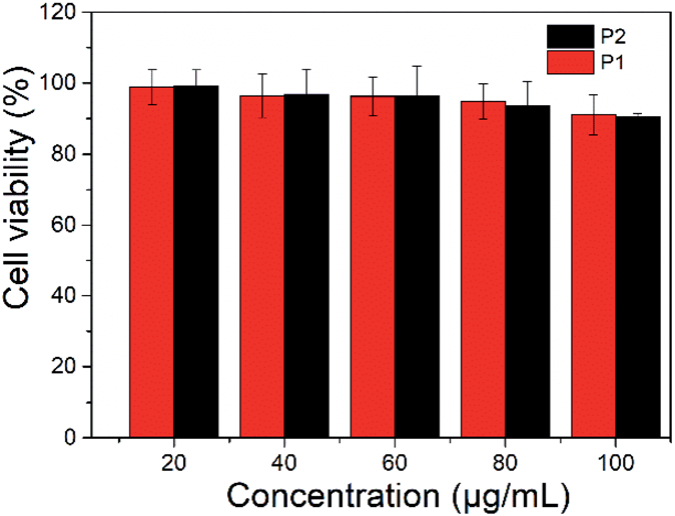
Cell viability of nanoparticles of P1 and P2.

The toxicity of polymer nanoparticles is an essential prerequisite in the field of biological diagnosis and treatment.^37^ Bcap 37 cells were employed to evaluate the cytotoxicity of P1 and P2 nanoparticles. A series of gradient concentrations of nanoparticles solutions were used to test their cytotoxicity and the MTT (3-(4,5-dimethyl-2-thiazolyl)-2,5-diphenyl-2*H*-tetrazolium bromide) assay shows that cell viability was only slightly reduced as the concentrations increased (Fig. 5). More than 90% viability was maintained after incubation of Bcap 37 cells with P1 or P2 nanoparticles, even at concentration of 100 μg mL^−1^. The results indicated that the nanoparticles have no obvious toxicity.

### Fluorescent imaging

**Fig. 6:**
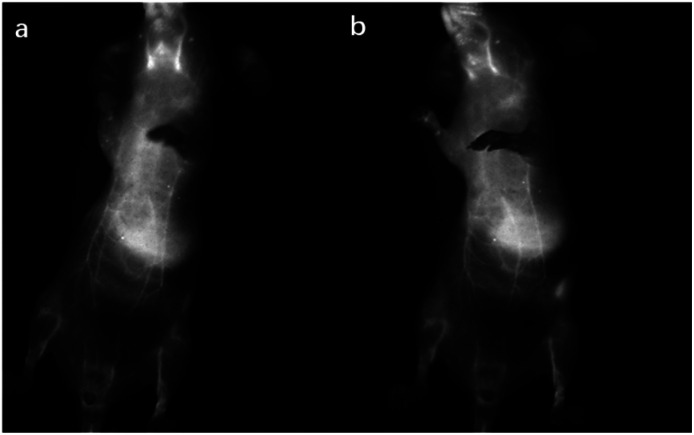
SWIR imaging of blood vessels in a nude mouse. Two minutes (a) and five minutes (b) after injection of nanoparticles of P2.

**Fig. 7:**
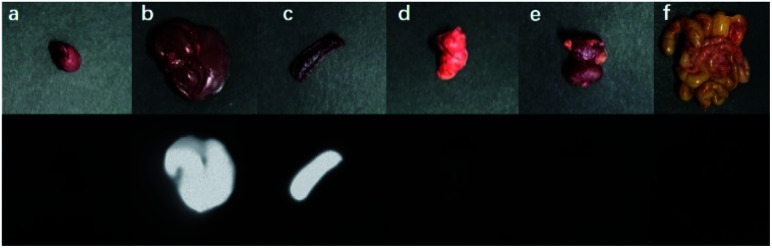
*Ex vitro* fluorescence imaging of various viscera of a mouse: (a) heart, (b) liver, (c) spleen, (d) lungs, (e) kidneys and (f) intestine.

The nanoparticles of P2 were selected for *in vivo* SWIR whole-body imaging of healthy mice after injection of nanoparticles of P2 (200 μg mL^−1^, 50 μL) through the tail vein. An InGaAs camera was used to image the mice under 635 nm laser excitation. As shown in Fig. 6a, the blood vessels could be clearly discriminated after 2 min postinjection and part of the particles begin to accumulate in the intraepithelial tissues (liver, spleen, *etc.*). The aggregation of nanoparticles in the intraepithelial tissue increased gradually with blood circulation (Fig. 6b). In order to further understand the distribution of P2 nanoparticles in the mice, the mice were killed and their main organs were excised and imaged. As shown in Fig. 7, fluorescence is enhanced in the liver and spleen, which indicated that the nanoparticles were mainly captured and concentrated by the reticuloendothelial system and imaged them gradually. This is consistent with the results of *in vivo* imaging. Other mice injected with nanoparticles continued to feed for two months, no abnormalities or deaths were found, indicating that the nanoparticle has good biocompatibility and has the potential as a SWIR fluorescent imaging agent.

### Photothermal and photoacoustic property

**Fig. 8:**
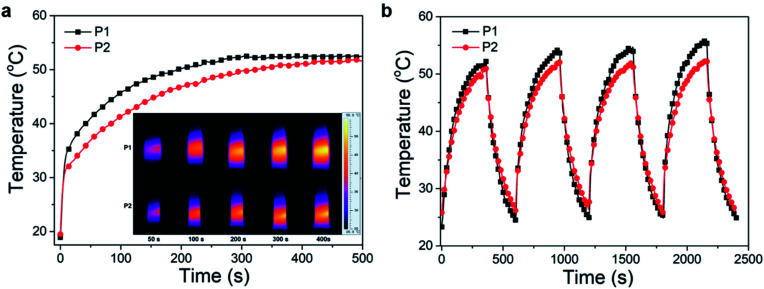
The temperature changes (a) and temperature stability (b) of P1, P2 nanoparticles solutions. The inset shows the NIR thermal images of P1, P2 nanoparticles solutions under NIR laser illumination (660 nm, 0.8 W cm^−2^).

The excitation energy absorbed by the conjugated polymer is mainly dissipated through two ways: (i) fluorescence emission and (ii) vibrational relaxation which lead to generation of heat.^31^ In other words, in addition to fluorescence, polymers may also have photothermal properties. Therefore, the photothermal properties of the nanoparticles solution were studied. An infrared thermometer was employed to monitor the temperature change of nanoparticles solution under continuous laser irradiation (Fig. 8a). The temperature of P1 and P2 nanoparticles solutions increased to 52 and 50 °C respectively after 400 s of 660 nm laser irradiation (0.8 W cm^−2^). This temperature is enough to kill cancer cells.^37^ The photothermal stability was investigated by cyclic photothermal heating and cooling processes, the maximum temperature of both solutions remained above 50 °C for at least 4 cycles (Fig. 8b), which indicated that both P1 and P2 nanoparticles have good light stability. This result is consistent with the fluorescence stability test.

**Fig. 9:**
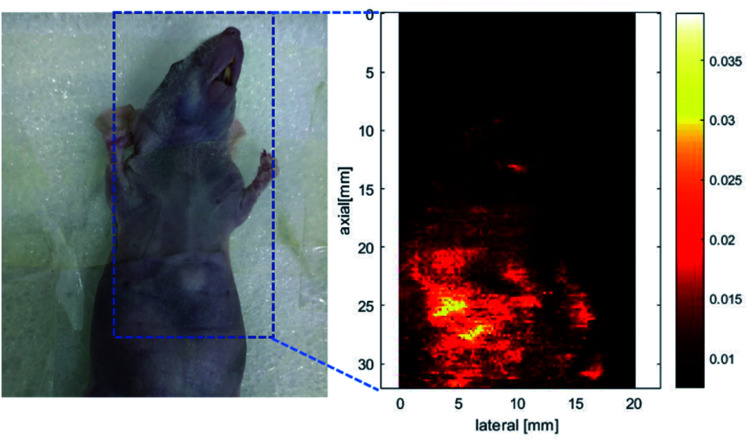
Photoacoustic imaging of a nude mouse. (a) A mouse used for photoacoustic imaging, in which the blue dashed box indicates the location of mouse photoacoustic imaging. (b) Photoacoustic imaging of the nude mouse in the blue dashed line.

Encouraged by the good photothermal properties of the nanoparticles, the photoacoustic properties of P2 nanoparticles were studied by a custom-built photoacoustic imaging setup with a 530 nm laser as excitation light. As can be seen in Fig. 9, after injection of P2 nanoparticles obvious photoacoustic signals can be observed in the liver. P2 nanoparticles has excellent short wave near infrared fluorescence, photoacoustic, and photothermal properties, which makes it possible to be used as an integrated reagent for diagnosis and treatment.

## Conclusions

In summary, donor unit with sp^3^ hybrid configuration and acceptor unit with small hindered alkyl side chain are employed to construct donor–acceptor (D–A) type conjugated polymers P1 and P2, which were substituted with one or two fluorine atoms. P1 and P2 nanoparticles show similar absorption bands in aqueous solution with major absorption peaks at 663 and 645 nm, respectively. And they showed an emission peak at about 945 nm with a tail extended to 1200 nm. Donor unit with sp^3^ hybrid configuration alleviate the aggregation-caused quenching, acceptor unit with small hindered alkyl side chain and fluorine substitute contribute to charge transfer, as a result the fluorescence quantum efficiency improved. The SWIR fluorescent quantum efficiency of P2 nanoparticles is 4.4%, which is one of the highest for organic SWIR fluorophore reported so far. In addition to its outstanding fluorescence efficiency, the photothermal and photoacoustic properties of the polymer were also investigated. Preliminary SWIR imaging studies indicated the potential of P2 as a SWIR fluorophore with excellent performance and our design strategy is efficient in improving the fluorescent quantum efficiency. With the efficient tunability of their structure and optical properties by optimization structure design, conjugated polymers can become promising probes for biological imaging in the SWIR window.

## Experimental details

The details of synthetic procedure of polymers, preparation of nanoparticles, calculation of quantum yield, cytotoxicity test, SWIR fluorescence imaging and photoacoustic imaging of nude mice were described in the ESI.†

## Ethical statement

All experiments were performed in compliance with the relevant laws of China. All animal procedures were performed in accordance with the Guidelines for Care and Use of Laboratory Animals of Qingdao University of Technology and approved by the Animal Ethics Committee of Qingdao University of Technology.

## Conflicts of interest

There are no conflicts to declare.

## Supplementary Material

RA-011-D1RA05097D-s001
