# Supplementary material for: Dithieno[3,2-b:2′,3′-d]silole-based conjugated polymers for bioimaging in the short-wave infrared region
Source: RSC Adv. 2021 Sep 16;11(49):30798–804. doi: 10.1039/d1ra05097d (PMC9041370; doi:10.1039/d1ra05097d)
Supplement: RA-011-D1RA05097D-s001 [file RA-011-D1RA05097D-s001.pdf]

Electronic Supplementary Material (ESI) for RSC Advances.
This journal is © The Royal Society of Chemistry 2021

Electronic Supplementary Information

Dithieno[3,2-b:2',3'-d]silole-based Conjugated Polymers for Bioimaging in Short-Wave Infrared Region

Chuantao Gu*[a,b], Haicheng Wang[a], Xiaoxia Wang[d], Shuguang Wen[b], Xiaoguang Liu[a], Weiqiang Tan[a], Meng Qiu*[c], Jiping Ma[a]

a. School of Environmental and Municipal Engineering, Qingdao University of Technology, Qingdao 266525, P. R. China.

b. CAS Key Laboratory of Bio-based Materials, Qingdao Institute of Bioenergy and Bioprocess Technology, Chinese Academy of Sciences, Qingdao 266101, P. R. China.

c. Key Laboratory of Marine Chemistry Theory and Technology (Ocean University of China), Ministry of Education, Qingdao266011, P. R. China

d. Qing Dao Municipal Hospital, Qingdao266011, P. R. China

E-mail: guchuantao@qut.edu.cn, mengqiu@ouc.edu.cn

Tel: +86-532-85071673

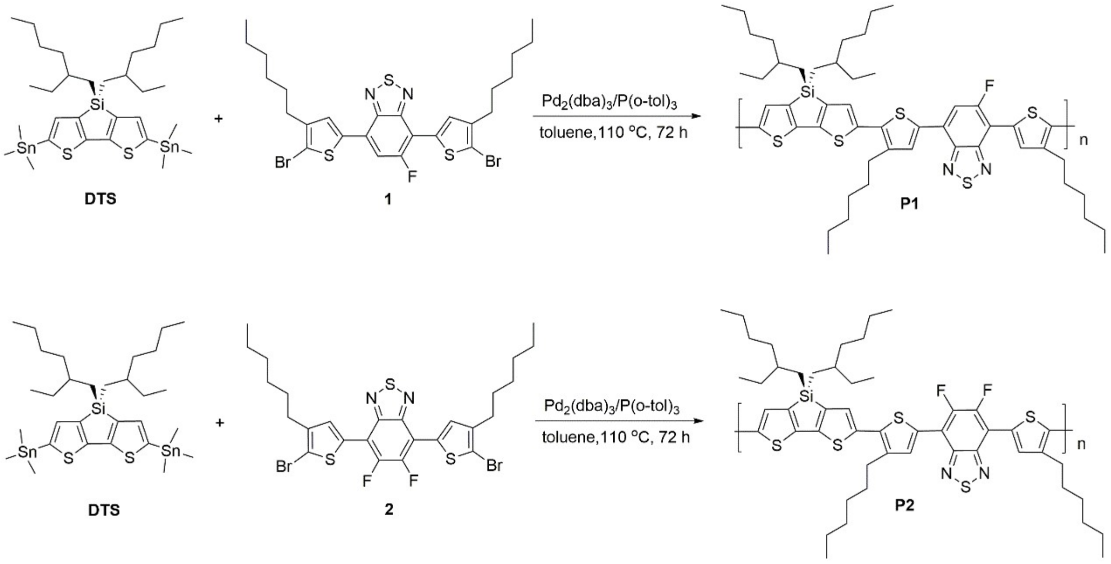

Scheme S1. Synthetic route of donor unit

1. General Synthetic Procedure of Polymers

Compound **DTS**[1], **1**[2] and **2**[3] were synthesized according to previously reported methods. The polymers were prepared by the same procedure through the Stille coupling reaction. In a 25 mL round-bottom flask, **DTS** (0.38 mmole), compound **1** (or **2**) (0.38 mmole), tris(dibenzylideneacetone)dipalladium (10 mg) and tris(*o*-tolyl)phosphine (25 mg) were subjected to three cycles of evacuation/nitrogen purging and then 8 mL of anhydrous toluene was added. The oil bath was heated to 110 °C slowly, and the reactant was stirred for 72 h at 110 °C under nitrogen atmosphere. The reaction mixture was cooled down to room temperature and precipitated in 150 mL of methanol. The precipitate was filtered then purified by Soxhlet extraction with methanol, hexane, and $CHCl_3$ in succession. $CHCl_3$ fractions were collected, concentrated, reprecipitated in methanol, and dried under vacuum overnight to give the target polymers.

P1: the polymer was obtained as a blue solid (223.7 mg, yield 78.0%). ^1H NMR (600 MHz, $CDCl_3$): δ (ppm) 8.12 (br, 1H), 7.98 (br, 1H), 7.71 (br, 1H), 7.22 (br, 2H), 2.87 (br, 4H), 1.76 (br, 4H), 1.53-1.16 (br, 34H), 0.98-0.82 (br, 18H).

P2: the polymer was obtained as a dark blue solid (243.6 mg, yield 81.2%). ^{1}H NMR (600 MHz, $CDCl_3$): δ (ppm) 8.10 (br, 2H), 7.20 (br, 2H), 2.88 (br, 4H), 1.76 (br, 4H), 1.50-1.14 (br, 34H), 1.00-0.80 (br, 18H).

2. Preparation of nanoparticles

The schematic illustration of nanoprecipitation was shown in Fig. S1. Nanoparticles were prepared by nanoprecipitation. 2 mg of P1 (or P2) and 10 mg of DSPE-mPEG2000 were dissolved in 8 mL of tetrahydrofuran, and the solution was quickly added to 72 mL of distilled water under strong ultrasonication, followed by distillation under reduced pressure to remove tetrahydrofuran and most of the water to obtain 4 mL of a concentrated nanoparticles solution at a concentration of 500 $\mu g \cdot mL^{-1}$.

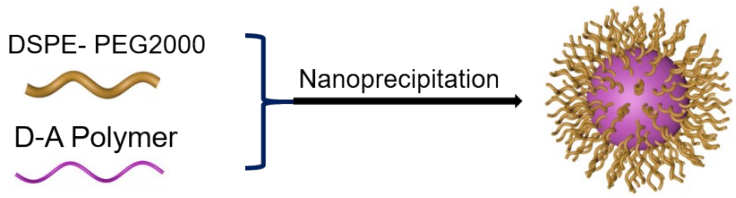

Fig. S1. Schematic illustration of nanoprecipitation

3. UV-vis absorption spectra of P1 and P2 in chloroform

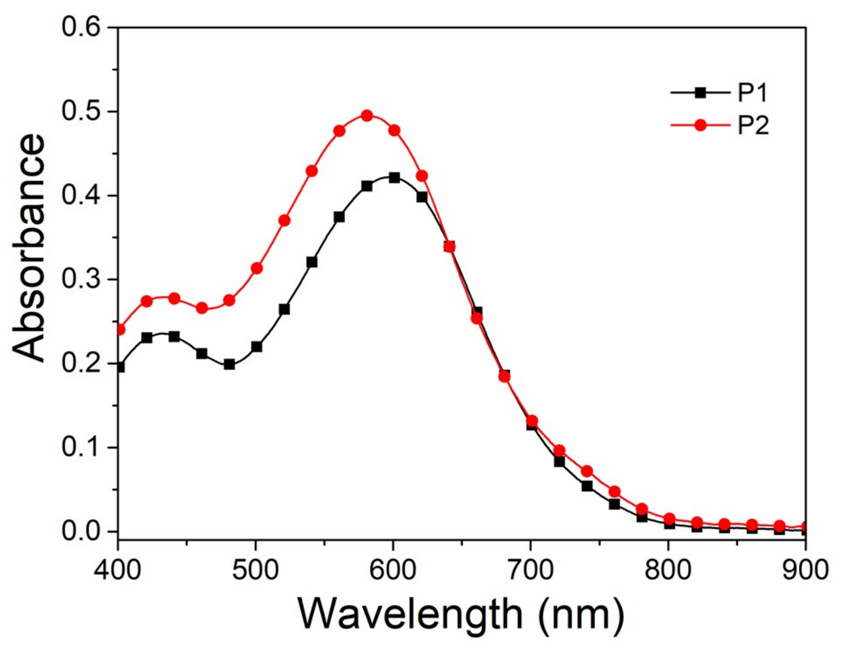

Fig. S2. Absorption spectra of P1 and P2 in $CHCl_3$ solution ($10^{-6} \cdot mol \cdot L^{-1}$)

4. Calculation of quantum yield[4]

IR-26 was used as a standard sample, and its absorption spectrum was measured by the UV-Vis-NIR to obtain five concentrations of solutions having absorbances at 808 nm of 0.023, 0.040, 0.053, 0.062, and 0.083. (Fig. S3a) The above five concentrations of IR-26 were excited by laser at 808 nm to obtain their fluorescence spectra (Fig. S3d). The range of 900-1500 nm in the fluorescence spectrum was integrated using Origin Lab, and the results were plotted according to the absorbance-integral area to obtain the intercept and slope of the curve (Fig. S3g).

Similarly, different concentrations of P1 and P2 nanoparticles solutions (Fig. S3b, c) were excited at 808 nm, and the above solkutions were excited by laser at 808 nm to obtain their fluorescence spectra (Fig. S3e, f). The range of 900-1500 nm in the fluorescence spectrum was integrated using Origin Lab, and the results were plotted according to the absorbance-integral area to obtain the intercept and slope of the curve (Fig. S3h, i).

Quantum yield is calculated by the following formula:

$$QY_{sample} = QY_{IR26} \times \frac{Slope_{sample}}{Slope_{IR26}} \times \frac{{n_{sample}}^2}{{n_{IR26}}^2}$$

Calculation result: QY_{P1}=3.4%, QY_{P2}=4.4%

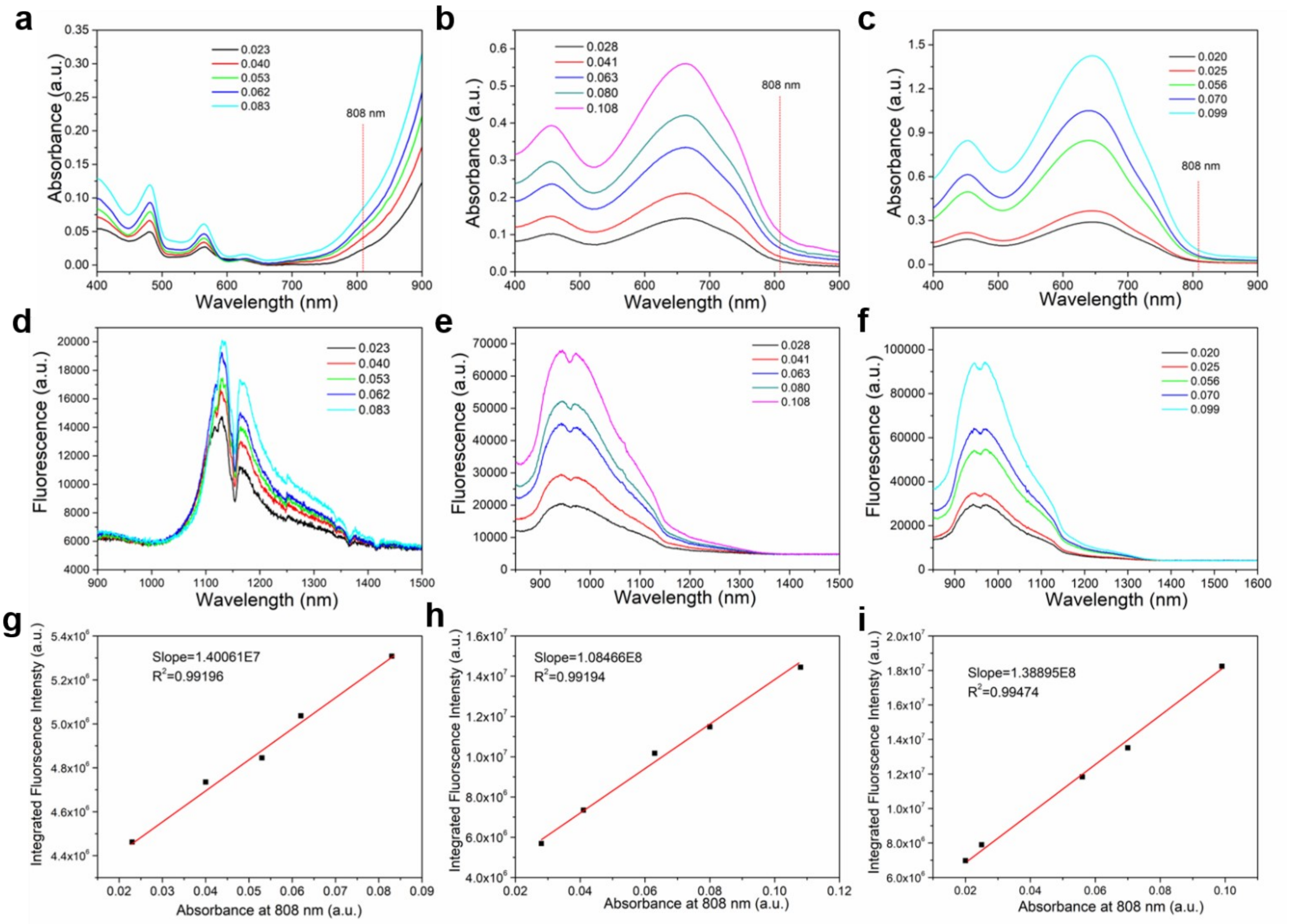

Fig. S3. a, b, c) Absorption spectra of IR26, P1 and P2; d, e, f) Fluorescence spectra of IR26, P1 and P2 excited at 808 nm; g, h, i) Fit curve of absorbance at 808 nm with integrated fluorescence intensity of IR26, P1 and P2.

5. Cytotoxicity test

Bcap 37 cells were used to test for cytotoxicity. The nanoparticles of P1 and P2 were dissolved in the culture medium at a concentration of 20 μg·mL^{-1}, 40 μg·mL^{-1}, 60 μg·mL^{-1}, 80 μg·mL^{-1} and 100 μg·mL^{-1}. Five concentrations of the solution were used to test cytotoxicity. Ten groups were tested in parallel for each concentration. After the highest and lowest values were removed, the average was obtained and the final result was obtained.

6. SWIR fluorescence imaging of nude mice

The nude mice used were 4 weeks old, and a laser of 635 nm was used as an

excitation light source. 50 μL of P2 nanoparticles solution (200 μg mL^{-1}) was injected into the nude mice. Nude mice were anesthetized with ether after injection of nanoparticles and then placed under a camera lens for imaging.

7. Photoacoustic imaging of nude mice

The nude mice used were 4 weeks old, the pulsed laser had a wavelength of 530 nm, the power of the laser is 1 W. The laser is irradiated within a circle with a radius of about 5 cm, and the laser intensity is about 1/(3.14*5*5)=0.013 W cm^{-2}. 50 μL of P2 nanoparticles solution (200 μg mL^{-1}) was injected into the nude mice. Nude mice were anesthetized with ether after injection of P2 nanoparticles solution, and then their limbs were fixed on a wooden board. The abdomen of the nude mouse is coated with ultrasonic glue, the ultrasonic glue is in contact with the lower surface of the plastic wrap, the water is directly contacted above the wrap film, and the signal receiver receives the acoustic signal in the water.

8. Measurements

A Bruker Advance III HD 400 (400 MHz) system was used to record the 1H NMR spectra of the organics. A Hitachi UH4100 system was used to record the UV-Vis-NIR absorption spectrum of the polymer at room temperature. An FLS980 system was used to record the fluorescence spectrum of the polymer. The photothermal data were measured by a FOTRIC 226 system. A Uninano-NIR II system was used for SWIR fluorescence imaging in nude mice.

Reference

1 J. Hou, H.-Y. Chen, S. Zhang, G. Li and Y. Yang, *J. Am. Chem. Soc.*, 2008, **130**, 16144-16145.

2 C. Gu, M. Xiao, X. Bao, L. Han, D. Zhu, N. Wang, S. Wen, W. Zhu and R. Yang, *Polym. Chem.*, 2014, **5**, 6551-6557.

3 S. Ko, E. T. Hoke, L. Pandey, S. Hong, R. Mondal, C. Risko, Y. Yi, R. Noriega, M. D. McGehee, J. L. Bredas, A. Salleo and Z. Bao, *J. Am. Chem. Soc.*, 2012, **134**, 5222-5232.

4 G. Hong, Y. Zou, L. A. Antaris, S. Diao, D. Wu, K. Cheng, X. Zhang, C. Chen, B. Liu, Y. He, J. Z. Wu, J. Yuan, B. Zhang, Z. Tao, C. Fukunaga and H. Dai, *Nat. Commun.*, 2014, **5**, 4206.
